# Effects of GLP-1 Receptor Agonists on Breast Reconstruction Outcomes: A Large-Database Retrospective Study

**DOI:** 10.3390/jcm15135042

**Published:** 2026-06-28

**Authors:** Bilal F. Hamzeh, Christopher R. Orear, Markos Mardourian, Carson Keeter, Katie G. Egan, Julian Winocour, George Kokosis, David W. Mathes, Christodoulos Kaoutzanis

**Affiliations:** 1 Division of Plastic and Reconstructive Surgery, Department of Surgery, University of Colorado Anschutz Medical Campus, Aurora, CO 80045, USA; bilal.hamzeh@cuanschutz.edu (B.F.H.); christopher.orear@cuanschutz.edu (C.R.O.); markos.mardourian@cuanschutz.edu (M.M.);; 2 Division of Plastic and Reconstructive Surgery, Rush University Medical Center, Chicago, IL 60612, USA

**Keywords:** breast reconstruction, implant-based reconstruction, glucagon-like peptide 1 receptor agonists, treatment failure, necrosis, postoperative complications

## Abstract

**Background/Objectives:** Glucagon-like peptide-1 receptor agonists (GLP-1RAs) are increasingly prescribed for diabetes and weight loss, with many breast reconstruction candidates being prescribed these medications. However, perioperative risks remain unclear. This study evaluated the association between GLP-1RA use and postoperative complications in implant-based and autologous tissue breast reconstruction. **Methods:** A retrospective analysis of TriNetX identified patients undergoing implant-based or autologous tissue breast reconstruction. Preoperative GLP-1RA users were compared to matched controls. Patients were propensity score matched (1:1 and 1:3) for demographics and comorbidities including body-mass index and timing of reconstruction (delayed vs. immediate). Ninety-day outcomes were assessed using logistic regression. **Results:** Between 2014 and 2024, 57,987 patients were identified, of which 823 were GLP-1RA users. Of those users, 326 patients undergoing implant-based reconstruction and 51 patients undergoing autologous reconstruction were matched to controls. In implant-based cohorts, GLP-1RA use was associated with increased odds of implant failure (1:1 OR 1.70, 95% CI 1.18–2.45, *p* = 0.0046), wound healing complications (1:1 OR 1.90, *p* = 0.027), and higher readmission/ED utilization (1:1 OR 1.80, 95% CI 1.04–3.21, *p* = 0.040). No significant differences were observed for hematoma, seroma, or thromboembolism. In autologous reconstruction, GLP-1RA use was not associated with increased risks. **Conclusions:** GLP-1RA use is linked to higher rates of implant failure, wound healing complications, and readmission in implant-based breast reconstruction only. These findings highlight the need for risk stratification and counseling of GLP-1RA users undergoing implant-based procedures and for further research investigating the implications of perioperative use of these agents in plastic surgery.

## 1. Introduction

Glucagon-like peptide-1 receptor agonists (GLP-1RAs) such as semaglutide and liraglutide, originally indicated for the treatment of type 2 diabetes mellitus, have garnered significant attention in recent years due to their ability to promote significant weight loss and morbidity reduction in overweight and obese patients [1,2]. Accordingly, skyrocketing demand for GLP-1RAs has resulted in a nationwide shortage [3]. Between January 2018 and June 2025, 2,196,747 patients received a total of 10,622,359 prescriptions for GLP-1RAs, with semaglutide (Ozempic^®^) being the most common first-time medication [4]. Recent studies discovered an increase in prescription dispensation of almost 600% between 2020 and 2023 and an increase in total spending on GLP-1RAs by over 500% between 2018 and 2023 [5,6]. Interestingly, current research is investigating the potential utility of GLP-1RAs in the treatment of diverse disease states including cardiovascular and renal disease, metabolic and gastrointestinal conditions, and for oncological indications including breast cancer [7,8,9,10,11,12].

These agents are, however, not without risks, especially in the perioperative period for plastic surgery. Although this class of medications has generally been viewed favorably by plastic surgeons, with almost 70% recommending their use to other plastic surgeons, complications associated with their use are still being elucidated [13,14]. Data suggest a potential association between GLP-1RA use and complications relating to skin laxity, volume loss, and body contour irregularities [15,16]. Other emerging evidence suggests possible associations between GLP-1RA use and postoperative complications in plastic surgery patients, with some cases highlighting a potentially increased risk of delayed wound healing, tissue fragility, and fat necrosis, specifically in breast surgery patients [17,18]. Even though guidelines for the perioperative use of GLP-1RAs have not yet been established in the context of plastic surgery, a recent Society for Perioperative Assessment and Quality Improvement (SPAQI) consensus suggests these agents are safe to continue with respect to the risk of anesthesia- and airway-related complications in surgery [19,20].

Female breast cancer is the most frequently diagnosed malignancy worldwide [21,22]. Among patients initially diagnosed with breast cancer in the United States, 33.4% were overweight and 33.8% were obese [23]. Furthermore, among women treated with prophylactic or oncologic mastectomy, 37.5% elect to undergo breast reconstruction surgery [24]. Given the prevalence of elevated body-mass index (BMI) in breast cancer patients, and considering that mastectomy and reconstruction is a cornerstone of breast cancer treatment, it is certain that surgeons will have to treat increasing numbers of patients on GLP-1RAs undergoing breast reconstruction. Therefore, it is imperative to understand the risk associated with perioperative GLP-1RA use among this population to better inform risk stratification.

There is a paucity of literature investigating the impact of GLP-1RA use on outcomes in plastic and reconstructive surgery overall, with data in breast reconstruction specifically being too limited still to support necessary formal guidelines and recommendations. In this study, we sought to analyze associations between preoperative GLP-1RA therapy and postoperative complication rates in patients undergoing implant-based and autologous tissue breast reconstruction to better understand the potential risks and enhance patient counseling and risk stratification among this rapidly growing population.

## 2. Materials and Methods

### 2.1. Data Collection

A retrospective analysis was performed using the TriNetX database (TriNetX LLC, Cambridge, MA, USA), a global federal research database of insurance claims data from more than 220 healthcare organizations (HCOs), mostly large, academic medical institutions, across over 30 countries [25]. The database is compliant with the Health Insurance Portability and Accountability Act (HIPAA) and maintains an Information Security Management System (ISMS) to protect and deidentify patients’ personal information [26]. Any data displayed on the TriNetX Platform in aggregate form, or any patient-level data provided in a dataset generated by the TriNetX Platform, only contain de-identified data as per the de-identification standard defined in Section §164.514(a) of the HIPAA Privacy Rule [27]. This study was exempt from Institutional Review Board approval as no protected health information was included in the database.

For this study, the TriNetX Network was queried to identify patients aged 18 years to 65 years who underwent either implant-based breast reconstruction including direct-to-implant (DTI) and tissue expander (TE) reconstruction or autologous tissue breast reconstruction including all flaps covered by the included Current Procedural Terminology (CPT) codes between May 2014 and May 2024 identified by CPT codes (Table S1).

### 2.2. Inclusion and Exclusion Criteria

Using TriNetX, patients were stratified into two groups: GLP-1RA users, defined by GLP-1RA prescription within 90 days preoperatively or postoperatively, and never GLP-1RA users. All patients who did not meet either criterion were excluded. Line-level data was downloaded from TriNetX, and data were subsequently analyzed in R version 4.4.1 (R Foundation for Statistical Computing, Vienna, Austria).

The dataset was split into two surgical groups: patients undergoing implant-based reconstruction (including DTI and TE patients) and patients undergoing autologous reconstruction (without filtering by flap type); each group was then further divided into a case and control group. The case group data was analyzed and subsequently stratified to only include patients with GLP-1RA prescription fills during the 90-day preoperative window using the brand name, RxNorm codes, or National Drug Codes (NDCs) (Table S2). Any case group patient for which a GLP-1RA medication could not be identified in this preoperative window was excluded. The case group and control group were further filtered to remove any patients with missing demographic information including age, sex, or BMI. It should be noted that prescription fills, as captured in claims-based databases, serve as a proxy for medication use and do not confirm actual adherence, perioperative discontinuation practices, specific dosing, or cumulative treatment duration.

### 2.3. Propensity Score Matching

Propensity score matching using 1:1 case–control greedy nearest-neighbor matching was performed in R Statistical Software. Caliper width was set to 0.2 to exclude any poorly matched case patients from further analysis. Covariates included in the propensity score matching model were age at time of surgery, sex, BMI, type 2 diabetes mellitus, hypertension, nicotine use status, chronic kidney disease, and surgical procedure by CPT code. Cohorts were also matched for timing of reconstruction (immediate vs. delayed reconstruction). Of note, if any of the above covariates were not present in any patients in the case group, all control group patients with said covariate were eliminated and the covariate was removed from the matching algorithm. Following propensity score matching, balance between the case and control groups was qualitatively evaluated using propensity score histograms and Love plots and quantitatively evaluated by comparison of group means and absolute standardized mean difference. An absolute standardized mean difference of <0.1 was considered indicative of acceptable covariate balance between groups. Subsequently, propensity score matching was repeated using a 1:3 case–control ratio to bolster statistical power using the excess amount of control patients and assess the robustness of the results across different matching ratios.

### 2.4. Outcomes

Following cohort identification and propensity-matching, 90-day postoperative outcomes were identified in each group and compared. Outcomes assessed included: infection, wound dehiscence, hematoma, seroma, grouped deep vein thrombosis (DVT) or pulmonary embolism (PE), grouped wound healing complications including necrosis and wound dehiscence, implant failure, emergency department (ED) visits, and hospital readmission, identified via International Classification of Diseases, Ninth/Tenth Revision, Clinical Modification (ICD-9/10 codes) and CPT codes (Table S3). Outcomes were grouped in cases where available ICD-9/10 codes could not be used to distinguish specific individual complications on TriNetX. Implant failure was identified using ICD-9/10 codes specific to breast implant complications and removal, supplemented by CPT codes for implant removal and replacement procedures (Table S3); however, the specific underlying cause of implant failure, including capsular contracture, implant rupture, or infection-driven explantation, cannot be distinguished at this level of coding specificity. A generalized linear model (logistic regression) was used to estimate the association between GLP-1RA use (case vs. control) and each outcome. In each case, the control group was used as the reference point. The regression model was fitted on the matched cohort, and odds ratios (ORs) with 95% confidence intervals (CIs) and *p*-values were reported.

### 2.5. Statistical Analysis

All statistical analysis was performed using R Statistical Software. Welch’s two sample *t*-test was used to compare group propensity score means. A *p*-value of <0.05 was determined to be statistically significant. Given the exploratory nature of this study, no formal correction for multiple comparisons was applied; findings should therefore be interpreted with appropriate caution, particularly those approaching the threshold for statistical significance.

## 3. Results

### 3.1. Cohort Identification

**Figure 1:**
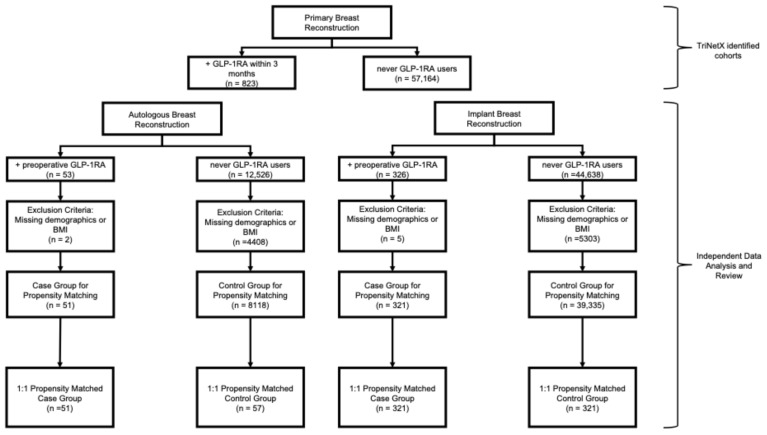
Flow diagram illustrating cohort identification, exclusion criteria, and propensity score matching workflow using TriNetX native analytics and independent investigator-led data review.

Between May 2014 and May 2024, a total of 57,987 patients aged 18 years to 65 years undergoing implant-based reconstruction or autologous breast reconstruction were identified on the TriNetX network, with 823 of these patients identified as perioperative GLP-1RA users. After application of exclusionary criteria, 51 patients were classified as preoperative GLP-1RA users undergoing autologous reconstruction and 326 patients were classified as preoperative GLP-1RA users undergoing implant-based reconstruction. Following propensity score matching, two balanced cohorts were obtained for each reconstructive modality (Figure 1).

### 3.2. Implant-Based Breast Reconstruction

#### 3.2.1. Propensity Score Matching, 1:1

**Figure 2:**
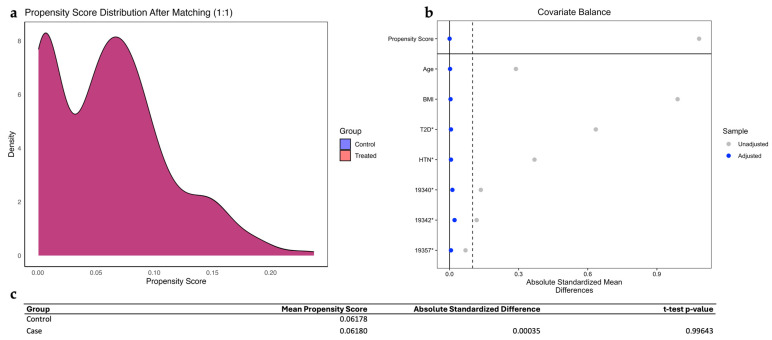
Propensity score matching parameters for 1:1 case–control implant-based breast reconstruction. (**a**) Propensity score distribution after 1:1 matching, demonstrating comparable distributions between case (red) and control (blue) cohorts (overlap between groups is represented as purple). (**b**) Covariate balance plot demonstrating absolute standardized mean differences before (gray) and after (blue) matching; all covariates fell below the 0.1 threshold following adjustment, indicating adequate balance. * indicates categorical variable. BMI = body mass index, T2D = Type 2 Diabetes Mellitus, HTN = hypertension, 19340 = code for immediate implant reconstruction, 19342 = code for delayed implant reconstruction, 19357 = code for tissue expander implantation. (**c**) Summary table of mean propensity scores, absolute standardized mean differences, and *t*-test *p*-values after matching, confirming no significant differences exist between groups.

Propensity score matching yielded well-balanced case and control cohorts for implant-based reconstruction with an absolute standardized mean difference < 0.1 across all covariates (Figure 2). Preoperative GLP-1RA use was significantly associated with an increased risk of 90-day complications including implant failure (OR 1.70, 95% CI 1.18–2.45, *p* = 0.0046), wound healing complications (OR 1.90, 95% CI 1.09–3.42, *p* = 0.027), and hospital readmission (OR 1.80, 95% CI 1.04–3.21, *p* = 0.040). No statistically significant differences were observed for hematoma, seroma, or thromboembolic events (Table 1).

#### 3.2.2. Propensity Score Matching, 1:3

Propensity score matching using 1:3 case–control ratios yielded well-balanced case and control cohorts for implant-based reconstruction (n = 1283) with absolute standardized mean differences < 0.1 across all covariates (Figure S1). Preoperative GLP-1RA use was significantly associated with an increased risk of 90-day complications including implant failure (OR 1.77, 95% CI 1.33–2.37, *p* = 0.00010), wound healing complications (OR 1.97, 95% CI 1.26–3.03, *p* = 0.024), and ED visit (OR 1.59, 95% CI 1.12–2.24, *p* = 0.0080). No statistically significant differences were observed for hematoma, seroma, or thromboembolic events (Table 2).

### 3.3. Autologous Tissue Breast Reconstruction

#### 3.3.1. Propensity Score Matching, 1:1

**Figure 3:**
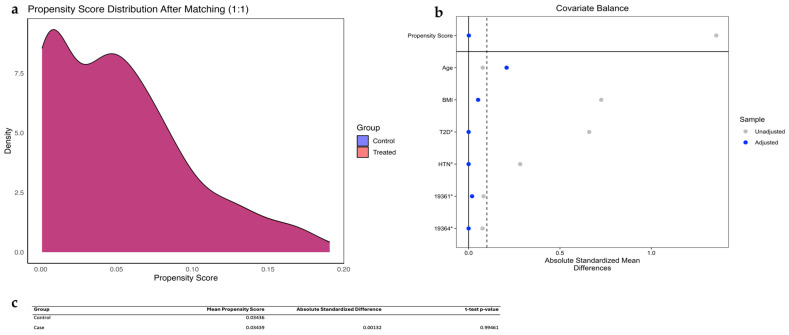
Propensity score matching parameters for 1:1 case–control autologous tissue breast reconstruction. (**a**) Propensity score distribution after 1:1 matching, demonstrating comparable distributions between case and control cohorts. (**b**) Covariate balance plot demonstrating absolute standardized mean differences before (gray) and after (blue) matching; all covariates fell below the 0.1 threshold following adjustment, indicating adequate balance. * indicates categorical variable. BMI = body mass index, T2D = Type 2 Diabetes Mellitus, HTN = hypertension, 19361 = code for reconstruction with latissimus dorsi flap, 19364 = reconstruction using a free flap. (**c**) Summary table of mean propensity scores, absolute standardized mean differences, and *t*-test *p*-values after matching, confirming no significant differences exist between groups.

Propensity score matching yielded well-balanced case and control cohorts for autologous tissue breast reconstruction with an absolute standardized mean difference < 0.1 across all covariates (Figure 3). Preoperative GLP-1RA use was not associated with an increased risk of any queried 90-day complications including wound healing complications, infection, or readmission (Table 3).

#### 3.3.2. Propensity Score Matching, 1:3

Propensity score matching using 1:3 case–control ratios yielded well-balanced case and control cohorts for autologous tissue breast reconstruction (n = 210) with an absolute standardized mean difference < 0.1 across all covariates (Figure S2). Preoperative GLP-1RA use was not associated with an increased risk of any queried 90-day complications including wound healing complications, infection, or readmission (Table 4).

## 4. Discussion

### 4.1. Interpretation of Findings

This is the first study to date, to the best of our knowledge, to investigate the association between GLP-1RAs and complications following both implant-based and autologous tissue breast reconstruction. We demonstrated a significant association between preoperative GLP-1RA use and increased postoperative complications in implant-based breast reconstruction, but there was no association between preoperative GLP-1RA use and 90-day postoperative complications in autologous tissue breast reconstruction. Among patients undergoing implant-based breast reconstruction with documented perioperative GLP-1RA use, the most salient findings included significantly increased risks of implant failure, wound healing complications, and hospital readmission. The plastic surgery literature offers mixed evidence relating to the effects of GLP-1RAs on postoperative outcomes. Notably, the only identified study directly investigating the effect of GLP-1RAs on outcomes in breast reconstruction also analyzed a large national cohort of 5618 patients who underwent DIEP flap reconstruction only. Three cohorts were propensity score matched: Group 1 (GLP-1RA with class I–II obesity), Group 2 (no GLP-1RA with class I–II obesity), and Group 3 (no GLP-1RA with class III obesity). The most salient finding showed that among patients with matched BMI, GLP-1RA use was associated with lower rates of revision surgery (5.7%, 12/211) compared to no GLP-1RA use (11.4%, 24/211) (*p* = 0.037) [28]. Lee et al. showed that patients taking GLP-1RAs, when compared to patients who did not, experienced a significantly increased rate of surgical site complications following panniculectomy (3.8% vs. 0.63%), abdominoplasty (5.56% vs. 0.15%), and, notably, breast reduction (1.92% vs. 0.66%) [29]. In addition, Taraschi and Salgarello reported on two patients who had surgery while on perioperative liraglutide; one patient underwent implant exchange with mastopexy, and the other patient underwent reduction mammoplasty. These patients both experienced wound dehiscence and fat necrosis, which aligns with our findings [18]. On the contrary, Albanese et al. compared outcomes in a prospective cohort of 21 patients undergoing circumferential lipoabdominoplasty who were taking GLP-1RAs for an average of 4.7 months (2–8 months) preoperatively against a control cohort of 21 patients without GLP-1RA use. While GLP-1RA use was associated with an increased mean incidence of hyperpigmentation (0.43 vs. 0.19; OR = 3.22; *p* = 0.10) and ecchymosis (0.43 vs. 0.15; OR = 4.27; *p* = 0.10), it was not associated with an increase in any major postoperative complications including venous thromboembolism, infections, necrosis, seroma or hematoma formation, or wound dehiscence following 360° lipoabdominoplasty [30].

Interestingly, we found no association between perioperative GLP-1RA use and postoperative complications in autologous tissue breast reconstruction, although this analysis was limited by a small sample size. The divergence between implant-based and autologous tissue breast reconstruction may reflect several underlying differences inherent to the procedures. While autologous tissue breast reconstruction such as deep inferior epigastric perforator (DIEP) flap reconstruction involves the transfer of abdominal tissue with robust blood supply, implant-based reconstruction following mastectomy relies on a skin flap for coverage of the device. One possible explanation, though speculative, is that GLP-1RA-related changes in tissue perfusion or wound healing may have a greater impact on mastectomy skin flaps covering implants than on well-vascularized autologous flaps; however, this hypothesis requires prospective validation and/or in vitro correlation. GLP-1RAs have been shown to alter tissue conditions, resulting in loss of dermal and subcutaneous adipose tissue and dysregulation of skin progenitor cells and hormonal and metabolic environments including influencing collagen production and skin regeneration [31,32]. The observation that perioperative GLP-1RA use does not appear to influence outcomes in autologous tissue breast reconstruction may therefore be a function of the intrinsic vascularity of the transferred tissue, greater tolerance to local skin conditions, and, potentially, the absence of a prosthetic foreign body; although given the observational nature of this study and the limited sample size in the autologous cohort, causal conclusions cannot be drawn. On the other hand, an animal study conducted by Zhu et al. showed that liraglutide can significantly enhance random skin flap viability and survival, which somewhat contradicts the above-mentioned theory. Liraglutide-mediated activation of transcription factor EB via the AMPK-MCOLN1-calcineurin signaling pathway was shown to enhance autophagy, prevent tissue oxidative stress, promote angiogenesis, and reduce pyroptosis, factors known to be crucial for flap survival [33]. While this model does not directly replicate free flap microvascular reconstruction, it suggests potential mechanisms by which GLP-1RA use may support, rather than impair, flap outcomes.

### 4.2. Limitations

This study faces limitations inherent to large-database retrospective studies. Due to the aggregate nature of data outputs in TriNetX, we were limited in our ability to analyze granular patient data. To overcome these limitations, it is essential to conduct large-scale retrospective or prospective cohort studies at single institutions or across many institutions to better assess the impacts of perioperative GLP-1RA use on outcomes in implant-based and autologous tissue breast reconstruction. Sample sizes also limited data analysis, particularly in the GLP-1RA autologous cohort, which consisted of 51 propensity-matched patients. The lack of significant findings within the autologous tissue breast reconstruction group should not be interpreted as evidence of a true absence of effect; with only 51 propensity-matched GLP-1RA users compared to a roughly 7-fold larger implant-based cohort, this analysis was underpowered, necessitating further research using adequately powered sample sizes. Moreover, the use of CPT and ICD codes are limited by individual provider- and institution-level billing practices and relies on the assumption that all events are coded correctly. CPT codes used to identify patients undergoing different breast reconstruction modalities may not capture a subset of patients undergoing procedures that were billed differently. In the case of specific complications, the use of ICD-9/10 codes also presented challenges. For example, some complications, such as seroma or hematoma, could not be stratified by donor or recipient site due to coding limitations. Moreover, specific wound healing complications such as mastectomy flap or nipple necrosis could not be distinguished using TriNetX, necessitating the use of grouped ICD codes which collectively represented wound healing complications. We were also unable to control for operative details including laterality, technique, adjunctive measures, mastectomy type (e.g., skin-sparing vs. nipple-sparing vs. oncologic breast reduction), or implant pocket plane (prepectoral vs. partial or complete subpectoral), all of which may influence reconstruction outcomes. Furthermore, patients with documented perioperative GLP-1RA prescription fills were assumed to be using their medication, but it is not possible to confirm adherence with this study design. Furthermore, the database does not permit granular assessment of GLP-1RA dosing regimens, dose titration schedules, or total treatment duration, all of which could plausibly influence complication risk and represent important variables for future investigation.

The use of large databases also cannot account for surgeon- and institution-specific factors including specific surgical techniques and perioperative management that may influence outcomes. While propensity score matching accounted for parameters such as surgery, sex, BMI, type 2 diabetes mellitus, hypertension, nicotine use status, and chronic kidney disease, it is possible that other parameters such as race or ethnicity, concomitant medications, history of neoadjuvant or adjuvant systemic therapies, and radiation history, in addition to other unmeasured comorbidities, represent residual confounders not fully addressed by propensity score matching. Nonetheless, the authors perceive this data as clinically valuable, particularly given the paucity of studies investigating the impacts of GLP-1RA use in the context of breast reconstruction. Future studies with granular patient demographic, comorbidity, and surgical data, in addition to more detailed information regarding the use and timing of GLP-1RA agents, are recommended to further elucidate the potential impacts of the medications on outcomes following breast reconstruction.

## 5. Conclusions

Understanding the risks associated with perioperative use of GLP-1RA agents in breast reconstruction is crucial given their skyrocketing popularity and diverse indications across aesthetic medicine, diabetes, and in managing weight. Our study shows the use of preoperative GLP-1RAs may be associated with an increased risk of postoperative complications including implant failure, wound healing complications, and readmission in implant-based breast reconstruction but no association with increased complications in autologous tissue breast reconstruction. Given the limitations associated with this database study, further research should prioritize institutional or multi-institutional retrospective and prospective studies with adequate sample sizes to further elucidate the potential impacts of these agents on breast reconstruction outcomes.

## Figures and Tables

**Table 1:** Postoperative complications following 1:1 propensity score–matched implant-based breast reconstruction (n = 642). Odds ratios (ORs) with 95% confidence intervals (CIs) and *p*-values are reported for matched case and control cohorts. DVT = deep vein thrombosis; PE = pulmonary embolism; ED = emergency department. * Denotes significance.

Complication	OR	95% CI Low	95% CI High	*p*-Value	Control Events	Case Events
Hemorrhage	5.36 × 10^7^	0.00	NA	0.997	0	1
Seroma	1.53	0.73	3.31	0.265	12	18
Hematoma	2.03	0.71	6.59	0.200	5	10
Infection	1.68	0.41	8.23	0.481	3	5
DVT or PE	5.36 × 10^7^	0.00	NA	0.997	0	1
**Implant Failure**	**1.70**	**1.18**	**2.45**	0.005 *	**63**	**94**
**Wound Healing Complications**	**1.90**	**1.09**	**3.42**	0.027 *	**20**	**36**
**Readmission**	**1.80**	**1.04**	**3.21**	0.040 *	**21**	**36**
ED Visit	1.51	0.98	2.34	0.064	41	58

* NA indicates a CI high unable to be calculated due to low number of events. Bolded lines indicate statistically significant differences between groups.

**Table 2:** Postoperative complications following 1:3 propensity score–matched implant-based breast reconstruction (n = 1283). Odds ratios (ORs) with 95% confidence intervals (CIs) and *p*-values are reported for matched case and control cohorts. DVT = deep vein thrombosis; PE = pulmonary embolism; ED = emergency department. * Denotes significance.

Complication	OR	95% CI Low	95% CI High	*p*-Value	Control Events	Case Events
Hemorrhage	1.46 × 10^8^	0.00	NA	0.996	0	1
Seroma	1.24	0.69	2.14	0.455	44	18
Hematoma	1.69	0.74	3.62	0.191	18	10
Infection	2.16	0.64	6.81	0.191	7	5
DVT or PE	1.50	0.07	15.71	0.741	2	1
**Implant Failure**	**1.77**	**1.33**	**2.37**	<0.001 *	**182**	**94**
**Wound Healing Complications**	**1.97**	**1.26**	**3.03**	0.002 *	**58**	**36**
Readmission	1.45	0.95	2.19	0.080	77	36
**ED Visit**	**1.59**	**1.12**	**2.24**	0.008 *	**117**	**58**

* NA indicates a CI high unable to be calculated due to low number of events. Bolded lines indicate statistically significant differences between groups.

**Table 3:** Postoperative complications following 1:1 propensity score–matched autologous tissue breast reconstruction (n = 106). Odds ratios (ORs) with 95% confidence intervals (CIs) and *p*-values are reported for matched case and control cohorts. DVT = deep vein thrombosis; PE = pulmonary embolism; ED = emergency department.

Complication	OR	95% CI Low	95% CI High	*p*-Value	Control Events	Case Events
Hemorrhage	NA	NA	NA	NA	0	0
Seroma	1.00	0.18	5.63	1.000	3	3
Hematoma	9.11 × 10^7^	0.00	NA	0.996	0	2
Infection	0.00	NA	Inf	0.996	1	0
DVT or PE	NA	NA	NA	NA	0	0
Wound Healing Complications	0.72	0.22	2.23	0.567	8	6
Readmission	0.52	0.20	1.30	0.167	15	9
ED Visit	0.72	0.22	2.23	0.567	8	6

NA indicates a CI high unable to be calculated due to low number of events.

**Table 4:** Postoperative complications following 1:3 propensity score–matched autologous tissue breast reconstruction (n = 210). Odds ratios (ORs) with 95% confidence intervals (CIs) and *p*-values are reported for matched case and control cohorts. DVT = deep vein thrombosis; PE = pulmonary embolism; ED = emergency department.

Complication	OR	95% CI Low	95% CI High	*p*-Value	Control Events	Case Events
Hemorrhage	NA	NA	NA	NA	0	0
Seroma	1.51	0.31	5.95	0.570	6	3
Hematoma	2.48 × 10^8^	0.00	NA	0.996	0	2
Infection	0.00	NA	6.90 × 10^171^	0.995	2	0
DVT or PE	NA	NA	NA	NA	0	0
Implant Failure	0.74	0.31	1.61	0.467	34	9
Wound Healing Complications	0.93	0.32	2.34	0.879	19	6
Readmission	0.98	0.41	2.19	0.971	27	9

NA indicates a CI high unable to be calculated due to low number of events.

## Data Availability

The data that support the findings of this study are available from TriNetX but restrictions apply to the availability of these data, which were used under license for the current study and are therefore not publicly available.

## Supplementary Materials

The following supporting information can be downloaded at: https://www.mdpi.com/article/10.3390/jcm15135042/s1, Table S1: CPT codes used to identify breast reconstruction operations. CPT = Current Procedural Terminology; TRAM = transverse rectus abdominis myocutaneous; DIEP = deep inferior epigastric perforator; SIEA = superficial inferior epigastric artery; SGAP = superior gluteal artery perforator; IGAP = inferior gluteal artery perforator.; Table S2. Brand names, NDC codes, and RxNorm codes used to confirm perioperative GLP-1RA use. NDC = National Drug Codes.; Table S3. ICD–9, ICD–10, and CPT codes used to identify 90-day postoperative complications. ICD–9/10 = International Classification of Diseases, Ninth/Tenth Revision; CPT = Current Procedural Terminology; DVT = deep vein thrombosis; PE = pulmonary embolism.; Figure S1. Propensity score matching parameters for 1:3 case-control implant-based breast reconstruction. (a) Propensity score distribution after 1:3 matching, demonstrating comparable distributions between case and control cohorts. (b) Covariate balance plot demonstrating absolute standardized mean differences before (grey) and after (blue) matching; all covariates fell below the 0.1 threshold following adjustment, indicating adequate balance. (c) Summary table of mean propensity scores, absolute standardized mean differences, and t-test p-values after matching, confirming no significant differences between groups.; Figure S2. Propensity score matching parameters for 1:3 case-control autologous tissue breast reconstruction. (a) Propensity score distribution after 1:3 matching, demonstrating comparable distributions between case and control cohorts. (b) Covariate balance plot demonstrating absolute standardized mean differences before (grey) and after (blue) matching; all covariates fell below the 0.1 threshold following adjustment, indicating adequate balance. (c) Summary table of mean propensity scores, absolute standardized mean differences, and t-test p-values after matching, confirming no significant differences between groups.
