# Supplementary material for: Effects of GLP-1 Receptor Agonists on Breast Reconstruction Outcomes: A Large-Database Retrospective Study
Source: J Clin Med. 2026 Jun 28;15(13):5042. doi: 10.3390/jcm15135042 (PMC13362899; doi:10.3390/jcm15135042)
Supplement: Supplementary file 1 [file jcm-15-05042-s001.zip › jcm-4367076-supplementary.pdf]

EFFECTS OF GLP-1 RECEPTOR AGONISTS ON BREAST RECONSTRUCTION OUTCOMES: A LARGE-DATABASE RETROSPECTIVE STUDY☆

SUPPLEMENTAL DIGITAL CONTENT • JOURNAL OF CLINICAL MEDICINE

Table S1. CPT codes used to identify breast reconstruction operations. CPT = Current Procedural Terminology; TRAM = transverse rectus abdominis myocutaneous; DIEP = deep inferior epigastric perforator; SIEA = superficial inferior epigastric artery; SGAP = superior gluteal artery perforator; IGAP = inferior gluteal artery perforator.

Category	CPT Code	Description
Autologous Breast Reconstruction	19361	Breast reconstruction with latissimus dorsi flap, with or without prosthetic implant
	19364	Breast reconstruction with free flap (e.g., free TRAM, DIEP, SIEA, SGAP, IGAP)
	19367	Breast reconstruction with pedicled TRAM flap
	19368	Breast reconstruction with bipedicle TRAM flap
	19369	Breast reconstruction with free TRAM flap
Implant-Based Breast Reconstruction	19340	Immediate breast reconstruction with implant
	19342	Delayed breast reconstruction with implant
	19357	Tissue expander placement for breast reconstruction (includes subsequent expansion)
Revision Breast Reconstruction	19380	Revision of reconstructed breast (includes adjustments to flap, implant, or symmetry procedures)

Brand Name	Generic Name	NDC Codes	RxNorm Code
Byetta	Exenatide	00002143380, 00002143401, 00002143480	310798
Bydureon / Bydureon BCise	Exenatide ER	00310654001, 00310654004, 00310654085	310798
Victoza	Liraglutide	00024574502, 00024574702, 00024576105	311093
Saxenda	Liraglutide	00024574902, 00024575102	311093
Trulicity	Dulaglutide	66780021007, 66780021008, 66780021201, 66780021904, 66780022601	311447
Ozempic	Semaglutide	00169280015, 00169291115	310713
Rybelsus	Semaglutide	00169406012, 00169406013	310713
Wegovy	Semaglutide	00169406112, 00169406113	310713
Tanzeum	Albiglutide	00310651201, 00310652004, 00310652401, 00310653001, 00310653004	312557
Adlyxin	Lixisenatide	00310652001, 00310652002	352160
Lixumia	Lixisenatide	—	352160
Mounjaro	Tirzepatide	00223500101, 00223500201, 00223500301, 00223500302	436363
Soliqua	Insulin Glargine + Lixisenatide	00604443501, 00604443502	316972
Xultophy	Insulin Degludec + Liraglutide	01690110512, 01690110524	440631

Table S2. Brand names, NDC codes, and RxNorm codes used to confirm perioperative GLP-1RA use. NDC = National Drug Codes.

Outcome	ICD-9 Codes	ICD-10 Codes	CPT Codes
Hemorrhage	998.11, 998.12	T81.000A–T81.099S	–
Seroma	998.13	L76.34	–
Hematoma	998.12	L76.32	–
Infection	998.5, 998.59	T81.400A–T81.409S	–
DVT or PE	451.1, 451.2, 451.81, 451.9, 453.4, 415.1	I82.40–I82.49, I82.20–I82.29, I82.90–I82.99, I26.0, I26.9	–
Implant Failure	996.54, 996.59	T85.41XA, T85.42XA, T85.43XA, T85.49XA, N65.0	–
Wound Healing Complications	998.83, 998.89, 998.9	T81.31XA, T81.89XA, L98.41	–
Readmission (Hospital)	–	–	99221, 99222, 99223, 99231, 99232, 99233, 99238, 99239
Emergency Department Visits	–	–	99281, 99282, 99283, 99284, 99285

Table S3. ICD–9, ICD–10, and CPT codes used to identify 90-day postoperative complications.
ICD–9/10 = International Classification of Diseases, Ninth/Tenth Revision; CPT = Current Procedural Terminology; DVT = deep vein thrombosis; PE = pulmonary embolism.

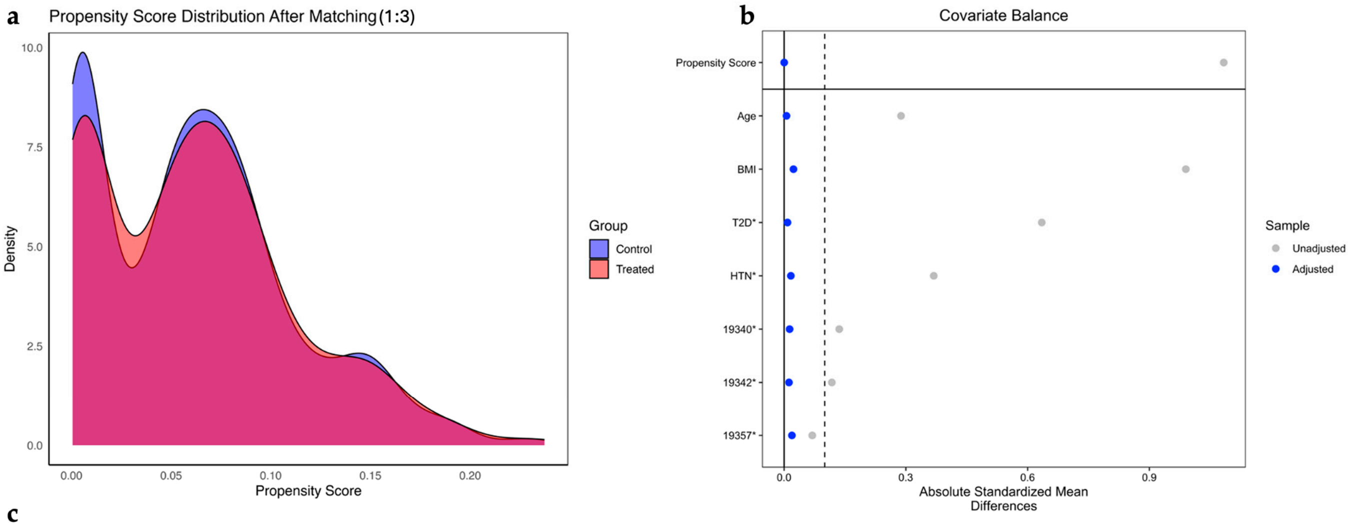

c

Group	Mean Propensity Score	Absolute Standardized Difference	t-test p-value
Control	0.06161		
Case	0.06180	0.00383	0.95270

Figure S1. Propensity score matching parameters for 1:3 case-control implant-based breast reconstruction. (a) Propensity score distribution after 1:3 matching, demonstrating comparable distributions between case and control cohorts. (b) Covariate balance plot demonstrating absolute standardized mean differences before (grey) and after (blue) matching; all covariates fell below the 0.1 threshold following adjustment, indicating adequate balance. (c) Summary table of mean propensity scores, absolute standardized mean differences, and t-test p-values after matching, confirming no significant differences between groups.

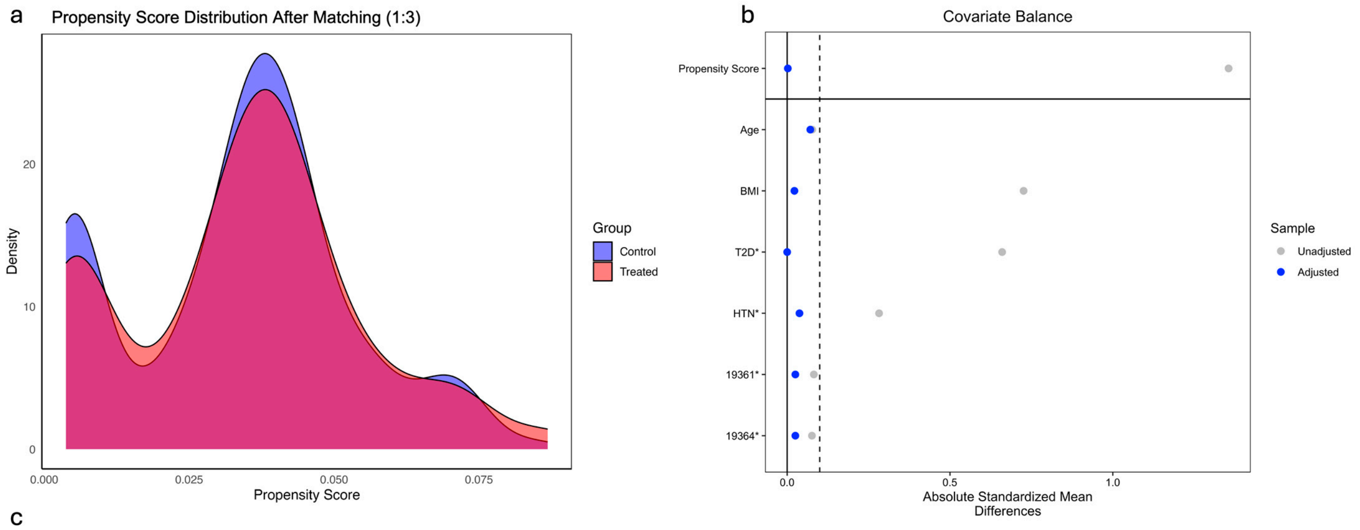

c

Group	Mean Propensity Score	Absolute Standardized Difference	t-test p-value
Control	0.03371		
Case	0.03439	0.03451	0.83059

Figure S2. Propensity score matching parameters for 1:3 case-control autologous tissue breast reconstruction. (a) Propensity score distribution after 1:3 matching, demonstrating comparable distributions between case and control cohorts. (b) Covariate balance plot demonstrating absolute standardized mean differences before (grey) and after (blue) matching; all covariates fell below the 0.1 threshold following adjustment, indicating adequate balance. (c) Summary table of mean propensity scores, absolute standardized mean differences, and t-test p-values after matching, confirming no significant differences between groups.
